# Biatrial Cardiac Metastases in a Patient with Uterine Cervix Malignant Melanoma

**DOI:** 10.1155/2015/958756

**Published:** 2015-04-28

**Authors:** Caglayan Geredeli, Melih Cem Boruban, Necdet Poyraz, Mehmet Artac, Alpay Aribas, Lokman Koral

**Affiliations:** ^1^Department of Medical Oncology, Konya Training and Research Hospital, 42090 Konya, Turkey; ^2^Department of Medical Oncology, Meram Medical Faculty, Necmettin Erbakan University, 42080 Konya, Turkey; ^3^Department of Radiology, Meram Medical Faculty, Necmettin Erbakan University, 42080 Konya, Turkey; ^4^Department of Cardiology, Meram Medical Faculty, Necmettin Erbakan University, 42080 Konya, Turkey

## Abstract

Primary malignant melanomas of uterine cervix are quite rarely seen neoplasms, and long-life prognosis of patients with this disease is poor. Immunohistochemical methods and exclusion of other primary melanoma sites are used to confirm the diagnosis. As with other melanomas, cervix malignant melanomas may also cause cardiac metastases. Cardiac metastases are among rarely seen but more commonly encountered cases, compared to primary cardiac tumors. Here, we present a case of biatrial cardiac metastases in a 73-year-old patient with uterine cervix malignant melanomas. The patient underwent echocardiography, cardiac magnetic resonance imaging, and computed tomography. Our report shows the importance of advanced diagnostic techniques, such as cardiac magnetic resonance, not only for the detection of cardiac masses, but for a better anatomic definition and tissue characterization. Although the cases of malignant melanomas leading to multiple cardiac metastasis were reported in literature, the metastatic concurrence of malignant melanomas in both right and left atriums is quite rarely encountered as metastatic malignant melanomas. Also, another intriguing point in our case is that the primary lesion of our case was stemmed from uterine cervix, but not skin.

## 1. Introduction

Malignant melanomas, common neoplasms of the mucous membranes and skin, constitute almost 2% of all newly diagnosed cancers [1]. Although malignant melanomas can occur in several mucosal sites, genital area in women is an extremely unusual involvement site. Among women, 3 to 7% of all cases are observed within genital tract, and the most witnessed sites are vulva and vagina. Primary malignant melanomas of uterine cervix are extremely rare neoplasms for which only 80 cases have been reported [2, 3].

Malignant melanomas are among the tumors with the highest rates of cardiac metastasis. However, cardiac metastases are diagnosed in about 1% of patients with malignant melanomas because nearly one-tenth of these patients present with cardiac symptoms [4]. The identification of cardiac metastases arising from melanomas usually demonstrates that systemic metastases are the reason of suffering in patients. In our report, we present a rare case with biatrial cardiac metastasis caused by uterine cervix malignant melanomas.

## 2. Case Presentation

**Figure 1:**
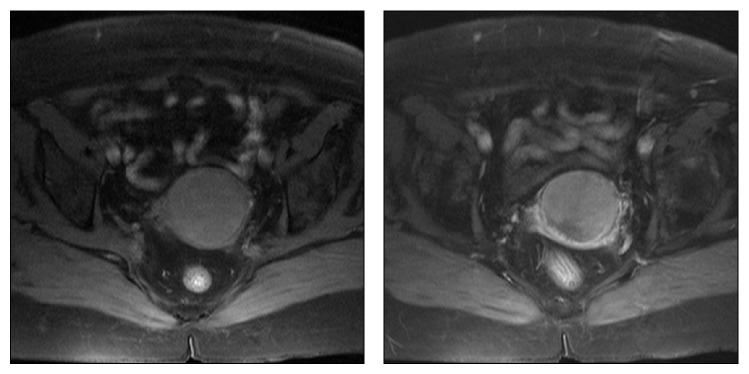
Pelvic MRI demonstrates a mass of malignant melanoma in size of 5.5 × 7.5 cm in uterine cervix area.

**Figure 2:**
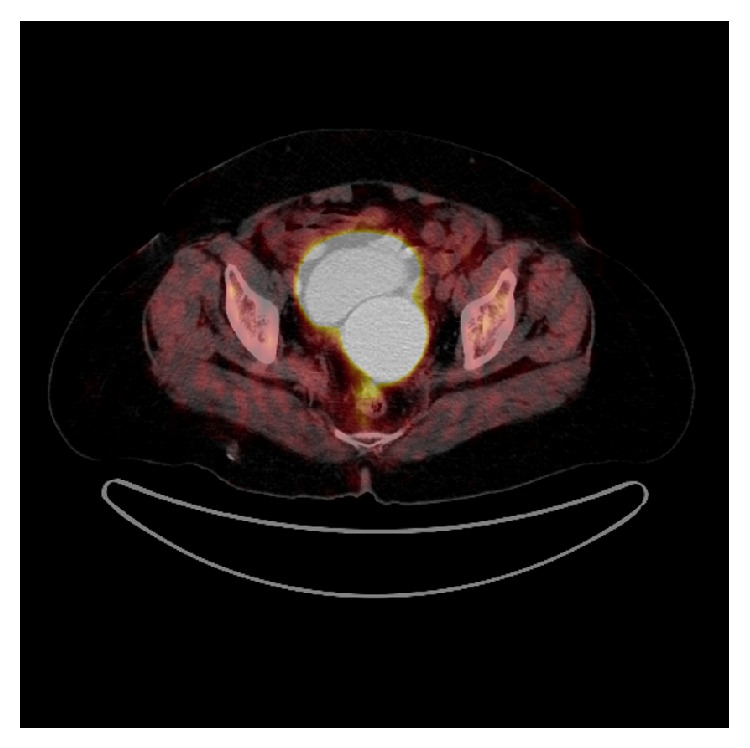
PET-CT demonstrates a mass of malignant melanoma in size of 65 × 63 × 98 mm with the uptake of fluoro-2-deoxy-D-glucose (FDG) (suv MAX 27.37) in uterine cervix area.

**Figure 3:**
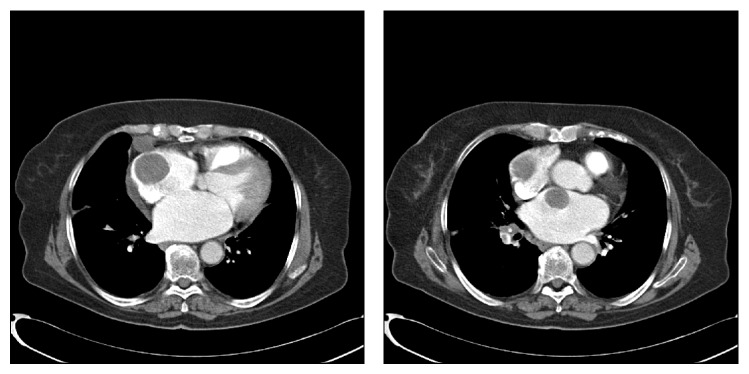
Thoracic CT demonstrates two masses with biatrial cardiac metastases of malignant melanomas in uterine cervix: one is 36 × 37 mm and the other is 26 × 20 mm in right and left atriums.

**Figure 4:**
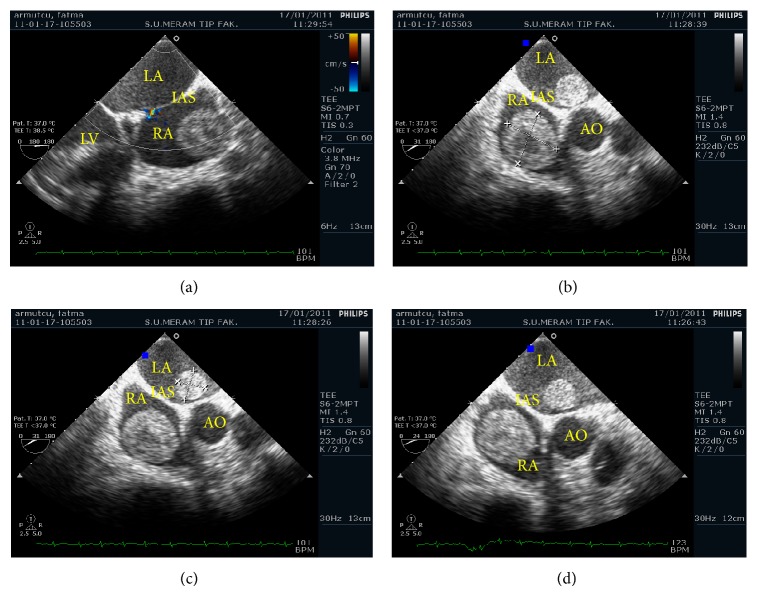
Transesophageal echocardiography demonstrates two mobile masses with biatrial cardiac metastases of malignant melanomas in uterine cervix, one attached to free wall (29 × 21 mm) in right atrium and the other attached to interatrial septum (20 × 17 mm) in the left.

**Figure 5:**
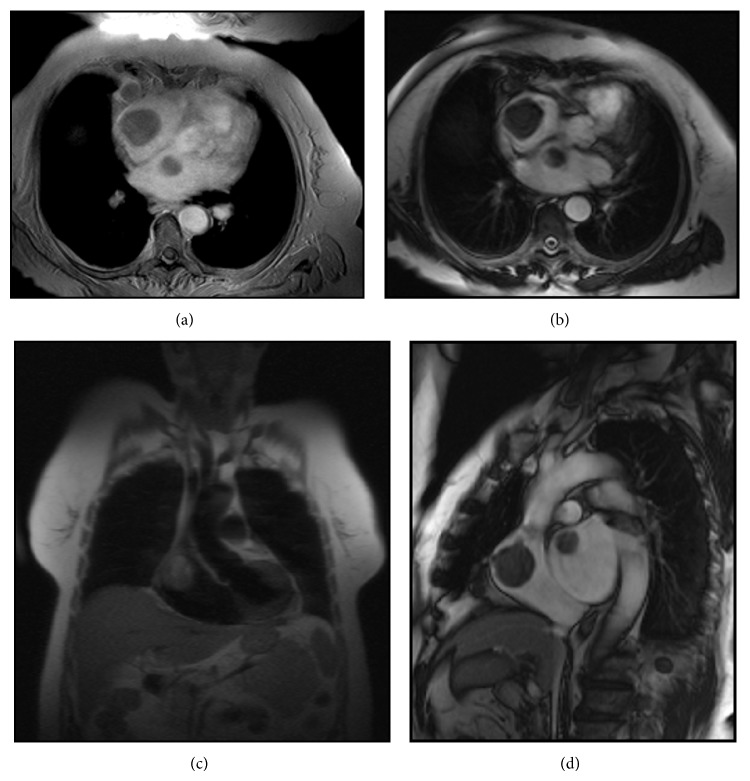
Cardiac MRI demonstrates two masses with biatrial cardiac metastases of malignant melanomas in uterine cervix: one is 37 mm and the other is 20 mm in right and left atriums.

A 73-year-old Caucasian woman had been admitted to the gynaecology and obstetrics department of our hospital with the complaint of vaginal bleeding. On the gynaecologic examination, a mass in size of nearly 5 cm was determined in uterine cervix. Magnetic resonance imaging (MRI) was performed, and a mass in size of 5.5 × 7.5 cm involving contrast material was detected in cervix area. The mass expanding into uteral parenchyma was seen to cause pressure on endometrium. Myometrium and uterine serosa were within normal limits (Figure 1). Positron emission tomography and computed tomography (PET-CT) revealed a mass of 65 × 63 × 98 mm with the uptake of fluoro-2-deoxy-D-glucose (FDG) (suv MAX 27.37) (Figure 2). The patient was surgically treated with type III hysterectomy + bilateral salpingooophorectomy + pelvic and para-aortic lymph node dissection + partial omentectomy. On pathologic examination, a mass of 4 × 3 × 1.8 cm immunohistochemically stained with HMB-45 and S-100 was detected and reported as malignant melanoma. No malignant melanoma metastases were witnessed in all of 38 resected lymph nodes. Within the postop period, the patient was referred to and followed up in the medical oncology department for the adjuvant therapy. Her initial blood pressure was 110/70 mm Hg, pulse rate 70 beats/min, respiratory rate 20/min, and body temperature 36.1°C. The general medical examination showed no pathology of respiratory, cardiovascular, or gastrointestinal systems. On the dermatologic investigation, no cutaneous lesion was observed in all parts of her skin. The treatment of adjuvant interferon alpha-2b (10 million IU/m^2^, three days a week) was commenced in the patient in whom no metastases were determined in any other parts of the body via PET-CT. On the thoracic CT performed four months after the initial treatment, two masses were seen, one in size of 36 × 37 mm in right atrium and the other in size of 26 × 20 mm in the left atrium (Figure 3). Via the transesophageal echocardiography, the images of two mobile masses were seen in each atrium, one attached to free wall in size of 29 × 21 mm in right atrium and the other attached to interatrial septum in size of 20 × 17 mm in the left atrium (Figure 4). Also, biatrial dilatation and ejection fraction were found as 55%. The images seen on CT and echocardiography were confirmed by performing cardiac MRI and commented as metastasis. Cardiac MRI demonstrates two masses with biatrial cardiac metastases of malignant melanomas in uterine cervix; one is 37 mm and the other is 20 mm in right and left atriums (Figure 5). Upon the evaluation as cardiac metastasis of uterine cervix malignant melanoma, systemic chemotherapy was administered, and the patient died three months after the initiation of systemic chemotherapy.

## 3. Discussion

Primary malignant melanomas of uterine cervix are difficultly diagnosed due to the lack of symptoms but may rarely lead to vaginal bleeding, as in our case. Meticulous examinations and detailed clinical investigations should be performed in order to diagnose correctly and early such an ailment with rare clues [5].

Malignant melanomas are known to show an aggressive biological behavior and a great tendency to cardiac metastases [6]. Most of such metastases occur after multifocal hematologic dissemination and may occur anywhere in the heart. Melanotic metastases can invade the walls of four cardiac chambers, and the right atrium is the most frequently involved site. Cardiac metastases typically involve the pericardium and myocardium while the endocardial layer is rarely involved. Additionally, metastatic melanomas of the pericardium and myocardium are usually multifocal lesions [7–9].

While cardiac metastases are the rarely seen initial manifestations, cardiac involvement usually occurs during the late stages of the disease. Therefore, the initial definitive antemortem diagnosis of metastatic melanomas of the heart is rare. Suspicious symptoms include otherwise-unexplained fever, heart murmurs, dysrhythmia, pericardial effusion, or heart failure [10–12].

CT or MRI may be beneficial in providing information, and PET is a noninvasive imaging technique used to detect occult or distant metastases at a relatively early stage and to clarify abnormal radiologic findings [10–12]. Cardiac metastases are seen as hypodense mass leading to filling defect after the injection of contrast material on CT images. A hypodense mass leading to filling defect in right atrium after contrast material injection is seen on our CT images as well (Figure 2). In the cardiac metastasis of malignant melanomas, hyperintense involvements are usually seen on T1-weighted cardiac MR images, heterogeneous hypointense involvements on T2-weighted images, and intense contrast material involvement after the injection of contrast material injection [13, 14]. In our cardiac MR images, heterogeneous hypointensity is seen on T2-weighted images, as well. An anatomic location of a tumor and the extent of invasion determine the feasibility of surgical intervention, which should optimally be performed during the early stages of the disease. Completely resecting an intracardiac melanoma prevents potential morbidities associated with progressive intracardiac growth, such as superior vena cava syndrome, right ventricular outflow and inflow obstruction, dysrhythmia, cardiac tamponade, and heart failure [10–12].

The medical therapy of patients with metastatic melanomas consists of the palliation of symptoms and systemic therapy with cytotoxic drugs, biotherapy, or immunotherapy. Long-term survival in these patients is associated with a complete response to systemic treatment. Although the cases of malignant melanomas leading to multiple cardiac metastasis were reported in literature [7], the metastatic concurrence of malignant melanomas in both right and left atriums is quite rarely encountered as metastatic malignant melanomas. Also, another intriguing point in our case is that the primary lesion of our case was stemmed from uterine cervix, but not skin.
